# Natural Ingredients to Enhance the Antioxidant Capacity in Different Meat Products: A Review

**DOI:** 10.3390/foods15050852

**Published:** 2026-03-03

**Authors:** Brisa del Mar Torres-Martínez, Armida Sánchez-Escalante, Gastón Ramón Torrescano-Urrutia, Rey David Vargas-Sánchez

**Affiliations:** Coordinación de Tecnología de Alimentos de Origen Animal (CTAOA), SECIHTI—Centro de Investigación en Alimentación y Desarrollo (CIAD), Carretera Gustavo Enrique Astiazarán Rosas 46, Hermosillo 83304, Mexico; brisa.torres@ciad.mx (B.d.M.T.-M.); armida-sanchez@ciad.mx (A.S.-E.); gtorrescano@ciad.mx (G.R.T.-U.)

**Keywords:** bioactive compounds, meat products, storage-gastrointestinal effect, antioxidant capacity

## Abstract

The oxidative stability of meat products is a crucial factor determining quality, shelf life, and consumer acceptance, as lipid and protein oxidation promote undesirable changes in sensory attributes and nutritional content. Antioxidant capacity (AOC) assays such as total phenolic content (TPC), ferric reducing antioxidant power (FRAP), 2,2′-azino-bis(3-ethylbenzothiazoline-6-sulfonic acid) (ABTS^•+^), and 2,2-diphenyl-1-picrylhydrazyl (DPPH^•^) are commonly applied in meat systems to assess the AOC associated with both intrinsic muscle components (endogenous) and the protective effects of natural ingredients (exogenous added compounds), i.e., antioxidants. Although differences in analytical methodologies limit direct comparisons among studies, it has been demonstrated that meat products inherently contain compounds that modulate oxidative reactions, with their effectiveness influenced by meat type, processing, and storage conditions. Within this framework, natural ingredients, including plant- and fungal-derived ingredients and their by-products, have gained attention as sources of natural antioxidants, whose capacity depends on the extraction method, the solvent used, and their behavior during gastrointestinal digestion, as evaluated using simulated gastrointestinal digestion (sGD) models. Numerous studies have shown that incorporating natural extracts or powders into meat products enhances AOC during refrigerated storage, with the effect generally depending on the concentration used. Moreover, several natural antioxidant treatments maintain or even enhance their AOC when assessed under sGD conditions.

## 1. Introduction

**Figure 1:**
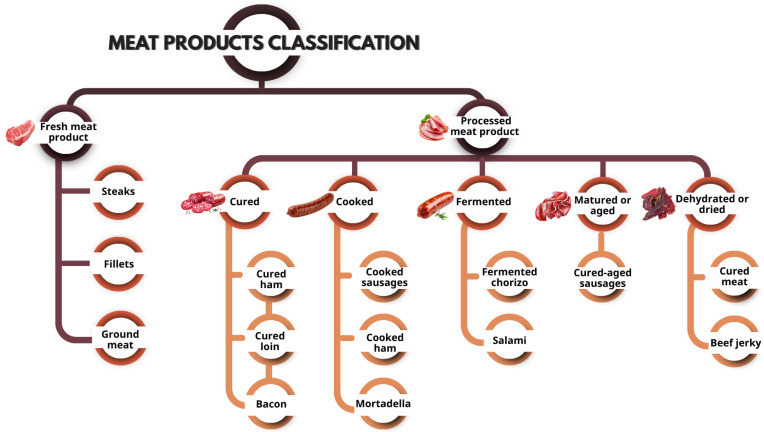
Meat product classification and representative examples.

Meat products are commonly classified by the type and intensity of processing applied (Figure 1). Fresh meat products consist of cuts or comminuted meat that have not undergone treatments beyond refrigeration or freezing. Additionally, processed meat products are characterized by the addition of ingredients and the application of physical, chemical, or biological treatments, which significantly modify their physicochemical and functional properties. This category includes cured products obtained through the use of curing salts and nitrates or nitrites; cooked products stabilized by heat treatment; fermented products preserved through controlled microbial activity; matured or aged products developed under defined conditions of time, temperature, and humidity; and dehydrated or dried products in which water removal contributes to shelf-life extension [1]. This classification is especially relevant when assessing oxidative stability and antioxidant strategies. The degree of processing strongly influences how lipids and proteins oxidize, as well as how bioactive compounds behave during storage and digestion [2]. It should be noted that meat product classification may vary across countries or regions, depending on the regulatory framework and technological criteria adopted.

Lipid and protein oxidation are among the primary challenges in preserving meat products, leading to the formation of potentially harmful compounds. For this reason, the food industry employs strategies to minimize oxidation and enhance the stability of meat products during processing and storage. Among these strategies, the use of natural antioxidants derived from plants and fungi, including their by-products, has received increasing attention due to their effectiveness and consumer acceptance of health, and these materials are recognized as rich sources of bioactive compounds, such as phenolics, flavonoids, and other secondary metabolites [3,4,5], which may contribute to enhance the oxidative stability of meat systems.

Advances in food chemistry and analytical methodologies have facilitated the evaluation of antioxidant capacity (AOC) through complementary in vitro assays (e.g., TPC, FRAP, ABTS^•+^, and DPPH^•^) and their application within complex matrices [4,5]. However, the antioxidant activity of these ingredients depends on multiple factors, including their chemical composition, extraction method, interactions with the meat matrix, processing conditions, and storage conditions. Furthermore, assessing their stability during storage and simulated gastrointestinal digestion (sGD) is essential to better understand their functionality and bioaccessibility, which are relevant beyond technological protection [3,6,7,8].

Based on the above, this review aimed to synthesize the available information on the use of natural ingredients, including plant- and fungal-derived ingredients and their byproducts, to enhance the AOC of meat products. Emphasis is placed on their analytical evaluation using antioxidant assays, their stability during processing and storage, and their behavior and functionality under sGD conditions.

## 2. Oxidative Stability of Meat Products

Lipid oxidation in meat products presents a significant challenge that extends beyond sensory deterioration, as it also compromises nutrient bioavailability and diminishes economic profitability. From a thermodynamic perspective, this process involves the activation of triplet oxygen into reactive species (O_2_, H_2_O_2_, O_2_^•−^, OH^•^) that can overcome the spin barrier and react with unsaturated fatty acids. In the initiation phase, hydrogen atom abstraction leads to the formation of alkyl radicals and conjugated dienes. This is followed by a lag phase, during which radical formation is limited by endogenous antioxidants in meat [9].

Protein oxidation represents another key pathway of quality deterioration and involves irreversible covalent modifications that affect the amino acid side chains, peptide backbone integrity, and the formation of intra- and intermolecular cross-links. These changes degrade the quality of meat products by altering texture, water-holding capacity, and functional behavior of proteins. Similar to lipid oxidation, protein oxidation proceeds through initiation, propagation, and termination phases mediated by reactive oxygen species (ROS), such as superoxide (O_2_^•−^), hydroperoxyl (HO_2_^•^), peroxyl (ROO^•^), and hydroxyl (OH^•^) radicals, which abstract hydrogen atoms to form carbon-centered radicals. Subsequent reactions with oxygen and transition metals promote the formation of peroxyalkyl radicals, alkyl peroxides, alkoxy radicals, and, under oxygen-depleted conditions, covalent cross-links that permanently modify the protein matrix [10].

**Figure 2:**
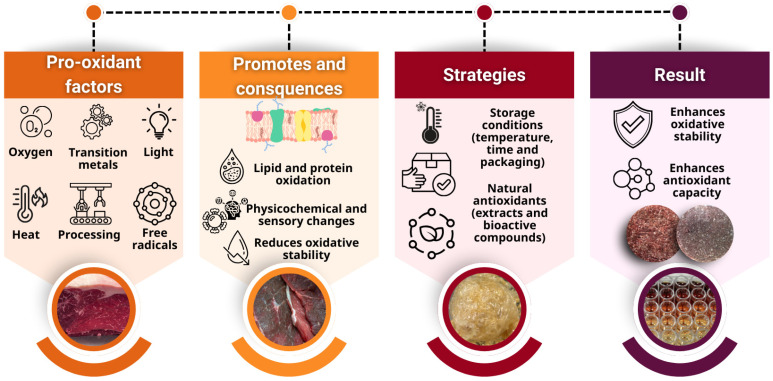
Schematic representation of factors affecting the oxidative stability of meat products.

As shown in Figure 2, oxidative stability is a crucial determinant of the quality, shelf life, and consumer acceptance of meat products, as lipid and protein oxidation lead to undesirable alterations in color, flavor, texture, and nutritional value. Several pro-oxidant factors contribute to the initiation and propagation of these reactions, including oxygen exposure, light, heat, processing conditions, free radical formation, and the catalytic activity of transition metals such as iron and copper, particularly in meat matrices rich in unsaturated fatty acids. Furthermore, technological variables such as the degree of processing, formulation ingredients, and storage conditions modulate oxidation kinetics [11,12,13,14,15].

**Figure 3:**
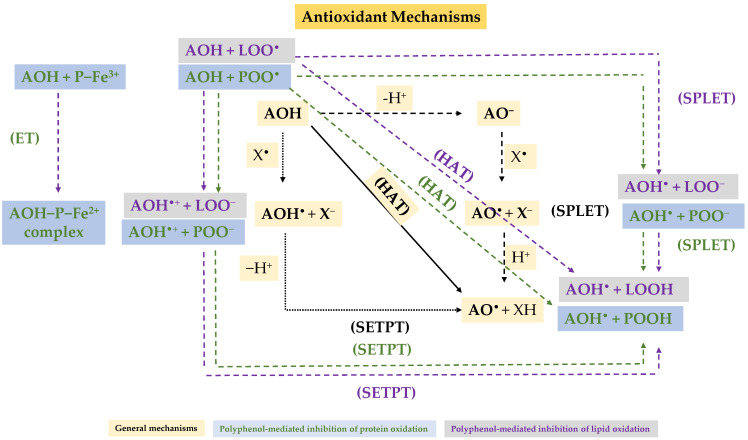
Schematic representation of some phenolic-antioxidant mechanisms in meat products. HAT, hydrogen atom transfer; SETPT, sequential electron−proton transfer; SPLET, sequential proton−electron transfer; ET, single electron transfer; AOH, phenolic antioxidant; AO^•^, phenoxyl radical; AOH^•^, phenol radical cation; LOOH, lipid hydroperoxide; POOH, protein hydroperoxide; LOO^•^, lipid peroxyl radical; POO^•^, protein peroxyl radical; P−Fe^3+^, protein−bound ferric ion; P−Fe^2+^, protein−bound ferreous ion.

Loss of oxidative stability accelerates physicochemical and sensory deterioration and promotes the formation of secondary oxidation products with potential health risks. To address this, strategies such as optimizing storage conditions and incorporating natural antioxidants have been widely studied to enhance the oxidative stability of meat products through different mechanisms (Figure 3) and to improve their AOC [16,17]. Assessing AOC with standardized analytical assays is essential for comparing the effectiveness of natural antioxidants and understanding their behavior during processing, storage, and digestion [18,19].

## 3. Analytical Assays for the Measurement of AOC in Meat Products

### 3.1. AOC Assays and Antioxidant Mechanism

The evaluation of AOC in meat and meat products poses challenges due to the diversity of available methods, with differences in their principles, experimental conditions, and reporting of results, making comparisons between studies difficult. Therefore, there is a need to standardize analytical procedures, including the extraction process, and to use combined methods to evaluate lipophilic and hydrophilic antioxidants to obtain a more complete profile [2].

The TPC is measured by the Folin–Ciocalteu method, which is based on electron transfer from the phenolic hydroxyl groups of the antioxidant to the Folin–Ciocalteu reagent (a mixture of phosphomolybdic and phosphotungstic acids). Under alkaline conditions, the reagent is reduced, forming a blue complex that is measurable spectrophotometrically at 760–765 nm. However, this method is not specific for polyphenols, as other reducing substances (e.g., ascorbic acid or reducing sugars) can also react [20,21,22].

Thus, the ferric reducing antioxidant power method (FRAP), which is also based on the electron transfer mechanism, measures the capacity of the antioxidant to reduce the ferric complex Fe^3+^−2,4,6-tris(2-pyridyl)-1,3,5-triazine acid (TPTZ) to its ferreous form Fe^2+^−TPTZ, which under acidic conditions forms a blue complex measured at 593 nm [23,24].

The radical cation inhibition has been extensively evaluated using the ABTS (2,2′-azino-bis(3-ethylbenzothiazoline-6-sulfonic acid)) method, which involves the generation of the ABTS^•+^ radical cation with an oxidizing agent, such as potassium persulfate. The process is based on an electron-transfer mechanism from the phenolic hydroxy groups of the antioxidant to the radical, leading to a decrease in the intensity of the blue-green color, which can be measured at 734 nm [24,25,26].

Additionally, the free radical inhibition has also been evaluated using the DPPH (2,2-diphenyl-1-picrylhydrazyl) method, which involves the hydrogen atom or electron transfer mechanism from the phenol hydroxylic groups of the antioxidant to the radical, resulting in a decrease in the intensity of the purple color, which is measured at 517 nm [26,27].

**Figure 4:**
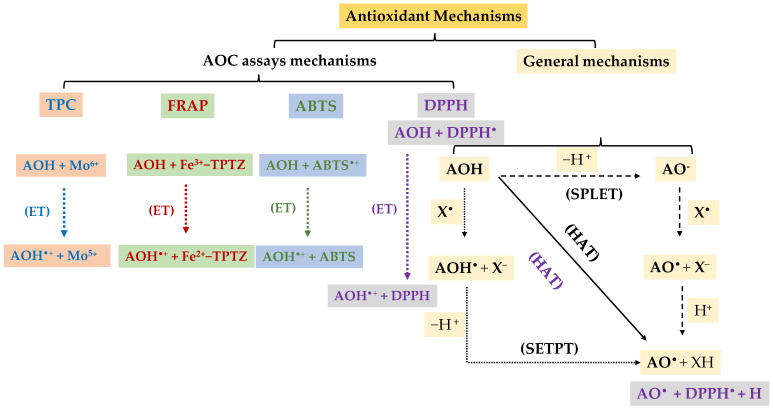
Schematic representation of some phenolic-antioxidant mechanisms. HAT, hydrogen atom transfer; SETPT, sequential electron-proton transfer; SPLET, sequential proton-electron transfer; ET, single electron transfer; AOH, phenolic antioxidant; AO^•^, phenoxyl radical; AOH^•^, phenol radical cation.

Although TPC, FRAP, ABTS, and DPPH assays differ in their experimental conditions, they are primarily based on electron- or hydrogen-atom-transfer mechanisms from phenolic hydroxyl groups to oxidizing species or free radicals. These reactions stabilize radical intermediates and interrupt oxidative chain reactions [20,21,22,23,24,25,26,27]. A generalized scheme of the radical-scavenging and metal-reducing mechanisms involved in AOC assays is illustrated in Figure 4, highlighting the fundamental role of phenolic structures in donating electrons or hydrogen atoms to neutralize reactive species.

### 3.2. Use of the AOC Assays in Meat and Meat Products

The application of antioxidant capacity (AOC) assays to meat and meat products without added antioxidants allows the evaluation of the intrinsic antioxidant potential of the meat matrix. This approach provides insight into the contribution of endogenous components, including enzymes, peptides, and micronutrients, to the modulation of lipid and protein oxidation. Furthermore, characterizing AOC establishes a reference framework for assessing the effectiveness of subsequent technological interventions or antioxidant additions, as well as for comparing oxidative behavior across different species and processing conditions [28].

Species-specific differences in endogenous AOC have been consistently reported. In poultry meat, hydrophilic extracts from breast and thigh muscles exhibited higher ABTS^•+^ radical scavenging activity than lipophilic fractions, highlighting the predominant contribution of water-soluble antioxidants in this matrix [29]. Similarly, endogenous AOC, as assessed by ABTS^•+^ and DPPH^•^ assays, has been documented in broiler breast meat, confirming the presence of intrinsic antioxidant systems [30].

In beef, aging processes significantly modulate AOC. Refrigerated aging has been associated with increased DPPH^•^ values, suggesting enhanced radical scavenging capacity over time [31]. Additionally, FRAP and DPPH^•^ activities have been detected in aged beef and were further influenced by subsequent heat treatment [32]. Comparative analyses across beef, pork, and poultry matrices also confirm measurable FRAP and ABTS^•+^ activities in fresh meat from different animal species, reinforcing the widespread presence of endogenous antioxidant compounds [33].

Thermal treatment represents a critical technological factor influencing endogenous antioxidant capacity, although its impact appears to be assay-dependent. In some cases, heating has been shown to enhance DPPH^•^ values, as reported for aged beef and dry-cured ham by-products, possibly due to the release of antioxidant peptides or Maillard reaction products with radical scavenging properties [32,34]. Conversely, cooking has been reported to reduce FRAP and ABTS^•+^ values in beef, pork, and chicken patties, indicating that processing intensity may degrade heat-sensitive antioxidant compounds or alter extraction efficiency [35]. These contrasting results emphasize that thermal effects on AOC depend on matrix composition, heating conditions, and the analytical method employed.

Beyond thermal treatments, other technological processes also influence endogenous AOC. Fermentation has been shown to increase ABTS^•+^ and DPPH^•^ values in chicken sausages compared to unfermented products, suggesting the generation of bioactive peptides or antioxidant metabolites during microbial activity [36]. Comprehensive evaluations of conventional meat products further indicate that endogenous total phenolic content (TPC) and AOC are generally low but highly variable depending on product type and processing intensity, among other factors. Reported TPC values in fresh and processed meats are typically limited, accompanied by modest FRAP activity and moderate ABTS^•+^ and DPPH^•^ radical inhibition, with significant variability among products [37].

Differences in reported AOC values across studies should not be attributed solely to species or processing, among other factors. Extraction protocols, solvent polarity, assay principles, and product formulation ingredients also influence measured inhibition percentages. Therefore, it is essential to consider both technological treatment and analytical methodology when evaluating and comparing AOC in meat systems. Incorporating natural antioxidant sources represents a targeted strategy to improve oxidative stability and enhance AOC in a controlled and reproducible manner. Understanding endogenous antioxidant systems and their modulation by processing provides a scientific basis for designing meat products with improved oxidative resistance.

## 4. Enhancing the AOC of Meat Products Using Natural Ingredients

### 4.1. Antioxidants of Natural Ingredients and Their Stability

Natural ingredients high in antioxidants are increasingly studied for their health benefits and use as natural food preservatives [38]. Studies have shown that natural ingredients derived from by-products such as peels, seeds, stems, and bagasse from fruits, vegetables, and grains possess significant AOC (Table 1).

These natural ingredients derived from by-products are typically processed by maceration, ultrasound-assisted extraction, or reflux, using water, ethanol, or hydroalcoholic solvents. Their AOC is commonly assessed through tests such as TPC, FRAP, ABTS^•+^, and DPPH^•^ assays. This bioactivity is largely associated with the presence of polyphenolic compounds, particularly flavonoids and phenolic acids, which act as hydrogen- or electron-donating antioxidants. The reported AOC varies widely depending on plant type and fungal material, extraction solvent, and processing conditions. Ethanolic and hydroalcoholic extracts commonly exhibit higher AOC than aqueous extracts, although matrix-dependent exceptions have been described. Among the evaluated natural ingredients derived from by-products, pomegranate, citrus, almond, pecan, and kiwi have consistently demonstrated high AOC, highlighting their suitability for food and nutraceutical applications.

Beyond extraction and processing, the gastrointestinal environment plays a critical role in determining the bioaccessibility and functionality of antioxidants derived from natural ingredients [55]. When evaluating sGD, differential effects on the TPC and AOC of plant matrices have been reported, depending on the plant source and pre-treatment method. For instance, Carneiro et al. [56] reported an increase in individual phenolic compounds in peel, seed, and oil fractions of *Schinus terebinthifolius* after sGD. Conversely, reductions in TPC and ABTS^•+^ values have been observed in lychee by-product, whereas increases in TPC were reported in jaboticaba. Similarly, decreases in FRAP, ABTS^•+^, and DPPH^•^ values have been reported for passion fruit peel and Puçá pulp, whereas carrot by-product and jujube peel exhibited enhanced AOC following digestion (Table 2). Collectively, these results demonstrate that sGD can either enhance or impair AOC, depending on matrix composition and solvent polarity. These variations are largely linked to the stability, release, and transformation of polyphenolic compounds, particularly flavonoids and phenolic acids, whose antioxidant activity depends on their chemical structures and interactions with the surrounding matrix during digestion.

### 4.2. AOC of Natural Ingredient-Treated Meat Products During Storage

AOC assays are widely used to evaluate the protective effect of natural or synthetic compounds against oxidative reactions in meat and meat products, which are among the main drivers of quality deterioration. These assays provide quantitative evidence of the efficacy of antioxidant ingredients, supporting the development of safer and more stable formulations and serving as essential tools for research and quality control in the meat industry.

Numerous studies have demonstrated that incorporating antioxidant-rich natural ingredients into meat formulations enhances meat AOC. Reyes-Padilla et al. [67] reported increased TPC and DPPH^•^ radical scavenging activity in Bologna-type mortadella formulated with combinations of pecan nut, prune, flaxseed, and cranberry. Similarly, Mancini et al. [68] showed that ginger powder improved FRAP, ABTS^•+^, and DPPH^•^ values in uncooked and cooked rabbit patties (depending on the concentration used), while garlic powder increased ABTS^•+^ values in rabbit patties without a clear dose–response assessment [69]. Researchers have found that adding citrus extracts to dry-cured sausages [70] and grape seed proanthocyanidins to lamb sausages improves AOC. These additions increased ABTS^•+^ and DPPH^•^ values compared to control samples [71].

In this regard, studies summarized in Table 3 indicate that uncooked meat products treated with natural ingredients, including plant- and fungal-derived ingredients and their by-products, consistently exhibit higher TPC, FRAP, ABTS^•+^, and DPPH^•^ values than untreated controls under storage conditions. This enhancement is primarily attributed to the presence of polyphenolic compounds, particularly flavonoids and phenolic acids, which contribute to antioxidant capacity via hydrogen-atom transfer and single-electron transfer mechanisms. The inclusion of plant- and fungal-derived materials has been shown to significantly enhance post-storage AOC. In some cases, the effect depends on the concentration used.

Additionally, studies summarized in Table 4 indicate that cooked meat products treated with natural ingredients, including plant- and fungal-derived ingredients and their by-products, consistently exhibit higher TPC, FRAP, ABTS^•+^, and DPPH^•^ values than untreated controls under storage conditions. This improvement is largely associated with the presence and thermal stability of polyphenolic compounds, particularly flavonoids and phenolic acids, which retain, or even enhance, their radical-scavenging activity after cooking due to structural transformations and interactions with the meat matrix. In some cases, the effect depended on the concentration used.

### 4.3. AOC of Natural Ingredient-Treated Meat Products Subjected to Gastrointestinal Digestion

The evaluation of AOC becomes particularly relevant in natural-treated meat products, as sGD can modify, release, or inactivate incorporated bioactive compounds [90]. In vitro gastrointestinal models offer a valuable tool for assessing the stability and bioavailability of antioxidants within complex meat matrices, providing insight into their potential health benefits and functional performance [91,92,93]. Several studies have demonstrated that sGD enhances the AOC of meat systems by releasing latent bioactive peptides and endogenous compounds. Carrillo et al. [32] found that sGD increased FRAP and ABTS^•+^ values in fresh and cooked meats from various animal species. Gallego et al. [33] and Wang et al. [94] also reported higher ABTS^•+^ and DPPH^•^ values in dry-cured pork products after digestion. Similarly, studies on cooked beef, pork, chicken, and venison showed that sGD raised FRAP, ABTS^•+^, and DPPH^•^ values, indicating improved antiradical activity [36,95].

The incorporation of natural antioxidant-rich ingredients into meat products further amplifies these effects during digestion. Biasi et al. [96] demonstrated that the addition of *Physalis peruviana* L. fruit flour to Bologna-type mortadella resulted in higher FRAP, ABTS^•+^, and DPPH^•^ values after sGD, with the effect depending on the concentration used. Similarly, Torres-Martínez et al. [97] reported that mushroom powder (*Pleurotus ostreatus*) enhanced TPC, TFC, FRAP, ABTS^•+^, and DPPH^•^ values in cooked pork patties following digestion (depending on the concentration used). Consistent with these results, studies summarized in Table 5 indicate that meat products formulated with mushroom extracts, vegetable oils, and fruit peels exhibit significantly higher AOC after sGD. This post-digestion enhancement is largely attributed to the release, transformation, and increased bioaccessibility of polyphenolic compounds, particularly flavonoids and phenolic acids, which may become more reactive under gastrointestinal pH conditions and exert antioxidant effects via hydrogen-atom transfer and single-electron transfer mechanisms. In several cases, higher inclusion concentrations of natural ingredients were associated with greater post-digestion AOC, demonstrating that these compounds can retain, or even enhance, their functionality under gastrointestinal conditions.

## 5. Conclusions

This review demonstrates that natural ingredients, including plant- and fungal-derived ingredients and their by-products, are an important source of bioactive compounds that enhance the antioxidant capacity (AOC) of meat products. The reviewed studies indicate that the AOC of these ingredients, as assessed by complementary analytical assays, is influenced by factors such as the origin of the raw material, extraction procedures, the characteristics of the meat matrix, and processing and storage conditions. Therefore, the combined use of different analytical methods is necessary to obtain a more comprehensive and reliable assessment of antioxidant behavior in meat systems.

Results derived from simulated gastrointestinal digestion (sGD) models suggest that many natural ingredients maintain or even increase their AOC after digestion.

However, several limitations must be acknowledged. The considerable heterogeneity in experimental designs, antioxidant sources, extraction techniques, meat formulations, and storage conditions limits direct comparisons between studies. Furthermore, most of the available evidence is based on in vitro chemical assays and sGD models, which may not fully reflect in vivo physiological conditions.

Therefore, future research should focus on establishing more harmonized methodologies for evaluating AOC in meat products and on better correlating chemical assays (TPC, FRAP, ABTS, and DPPH) with actual markers of oxidative stability.

Finally, the use of natural ingredients as antioxidant sources represents a promising strategy for improving the stability of meat products. Continued research in this area will support a more rigorous, evidence-based application of natural functional ingredients in the meat industry.

## Figures and Tables

**Table 1:** Natural ingredients and their AOC.

Reference	Natural Ingredient	Extraction of the Antioxidant Material	Bioactive Compounds Linked to AOC	Results of AOC
Selani et al. [39]	Grape (*Vitis labrusca*)	Material: seed and peel Method: maceration extraction (ø) Solvent: hydroalcoholic (80% EtOH; 48 h/25 °C) Separation: filtration (Whatman No. 1) Concentration: under vacuum at 65 °C	Flavonoids: (≠) Phenolic acids: (≠)	The extract exhibited high TPC
Packer et al. [40]	Beetroot (*Beta vulgaris* L.) Guava (*Psidium guajava* L.)	Material: stems and pomace, respectively Method: ultrasound-assisted extraction (15 min) Solvent: hydroalcoholic (80% ethanol) Separation: centrifugation (5000× *g*)	Flavonoids: epicatechin and quercetin Phenolic acid: 2,6-dihydroxybenzoic, isovanillic, syringic, p-coumaric, m-coumaric, gallic, ferulic, caffeic, and sinapic	The extract exhibited TPC, ABTS^•+^, and DPPH^•^ values (Beetroot stems > guava pomace)
Kim et al. [41]	Coffee (*Coffea* sp.)	Material: spent grounds Method: maceration extraction (ø) Solvent: H_2_O and EtOH-96% (1 h/80 °C) Concentration: under vacuum (ø)	Flavonoids: (≠) Phenolic acids: (≠)	The extract exhibited TPC and DPPH^•^ values (EtOH > H_2_O)
Turgut et al. [42]	Pomegranate (*Punica granatum* L.)	Material: peel Method: reflux-extraction (ø) Solvent: H_2_O (1:10 proportion) for 1 h Separation: filtration and centrifugation (1200× *g*/20 min) Concentration: under vacuum (40 °C) Drying: freeze-drying (0.120 mbar/72 h/−40 °C)	Flavonoids: (≠) Phenolic acids: (≠)	The extract exhibited TPC and DPPH^•^ values
Cruz-Trinidad et al. [43]	Mango (*Mangifera indica* L.)	Material: peel, seed, and paste Method: maceration extraction (ø) Solvent: H_2_O	Flavonoids: quercetin, gallocatechin, catechin, mangiferin, and gallocatechin gallate Phenolic acids: gallic and ellagic	The extract exhibited TPC, FRAP, ABTS^•+^, and DPPH^•^
Antonini et al. [6]	Chia (*Salvia hispanica* L.)	Material: seed Method: maceration extraction (ø) Solvent: hydroalcoholic solution (80% EtOH; 160 rpm/24 h/room temperature) Separation: centrifugation (3000 rpm/5 min)	Flavonoids: quantified (≠) Phenolic acids: (≠)	The extract exhibited TPC, ABTS^•+^, and DPPH^•^ values
Bedrníček et al. [44]	Onion (*Allium cepa* L.)	Material: peel Method: maceration extraction (ø) Solvent: hydroalcoholic (90% MeOH; 10 min) Separation: centrifugation (7000 rpm/15 min/5 °C)	Flavonoids: quercetin, Quercetin-4-O-glucoside, and Quercetin-3,4-O-diglucoside Phenolic acids: (≠)	The extract exhibited TPC, FRAP, and DPPH^•^ values
Villasante et al. [45]	Pecan (*Carya illinoinensis*)	Material: shell Method: ultrasound-assisted extraction (ø) Solvent: hydroalcoholic (50% EtOH; 30 min/50 °C)	Flavonoids: (≠) Phenolic acids: (≠)	The extract exhibited TPC and DPPH^•^ values
Barkhordari & Bazargani-Gilani [46]	Apple (*Malus domestica* L.)	Material: peel Method: ultrasound-assisted extraction (ø) Solvent: H_2_O and EtOH solution (96%; 20 kHz/30 min/25 °C) Separation: filtration and concentration (50 °C)	Flavonoids: (≠) Phenolic acids: (≠)	The extract exhibited DPPH^•^ activity (EtOH > H_2_O)
Boeira et al. [47]	Corn (*Zea mays* L.)	Material: stigma Method: ultrasound-assisted extraction (ø) Solvent: hydroalcoholic (70% EtOH; 20 kHz/5 min/60 °C) Separation: centrifugation (202× *g*/15 min) Concentration: under vacuum (45 °C)	Flavonoids: quantified (≠) Phenolic acids: (≠)	The extract exhibited TPC and DPPH^•^ values
de Santana Neto et al. [48]	Yellow mombin (*Spondias mombin* L.)	Material: bagasse (husk, seed, and residual pulp) Method: maceration extraction (ø) Solvent: hydroalcoholic (55% EtOH; 35 min/70 °C) Separation: centrifugation (8960 × *g*/10 min/10 °C) Concentration: under vacuum (180 mbar/45 °C)	Flavonoids: rutin, catechin, and myricetin Phenolic acids: gentisic, salicylic, hydroxybenzoic, ellagic, p-coumaric, ferulic, and caffeic	The extract exhibited TPC
Abdel-Naeem et al. [49]	Lemon (*Citrus limon*) Orange (*Citrus sinensis*) Grapefruit (*Citrus paradisi*) Banana (*Musa* spp.)	Material: peel Method: maceration extraction (ø) Solvent: hydroalcoholic (80% MetOH) Separation: centrifugation (10,000 rpm/15 min/4 °C)	Flavonoids: quantified (≠) Phenolic acids: (≠)	The extract exhibited TPC and DPPH^•^ (Lemon > Orange > Grapefruit > Banana)
Timón et al. [50]	Almond (*Prunus dulcis*)	Material: skin Method: maceration extraction (60 min/30 °C) Solvent: H_2_O (1:20 proportion) Separation: centrifugation (3000× *g*/10 min/4 °C)	Flavonoids: catechin, eriodictyol-7-O-glucoside, quercetin-3-O-rutinoside, Kaempferol-3-O-rutinoside, Isorhamnentin-3-O-rutinoside, and Kaempferol-3-O-glucoside Phenolic acids: protocatechuic, hydroxybenzoic, 4-coumaric	The extract exhibited TPC values (Antoñeta = Belona = Soleta ≥ Guara) The extract exhibited ABTS^•+^ and DPPH^•^ values (Antoñeta = Belona = Soleta ≥ Guara)
Cava & Ladero [51]	Pomegranate (*Punica granatum* L.)	Material: peel Method: maceration extraction (60 rpm/120 min/21 °C) Solvent: H_2_O, EtOH, and hydroalcoholic (50% EtOH)	Flavonoids: quantified (≠) Phenolic acids: (≠)	The extract exhibited TPC, FRAP, ABTS^•+^, and DPPH^•^ values (H_2_O > EtOH and H_2_O-EtOH)
Glišić et al. [52]	Sunflower (*Helianthus annuus* L.) Corn (*Zea mays* L.)	Material: stalk Method: reflux-extraction (ø) Solvent: hexane (1:6 proportion; 1 h/40 °C) Separation: filtration and centrifugation (ø) Concentration: under vacuum (60 rpm/150 min/60 °C).	Flavonoids: tricin Phenolic acids: p-coumaric and ferulic	Both extracts exhibited TPC, FRAP, ABTS^•+^, and DPPH^•^ values
Xu et al. [53]	Citrus (*Citrus sinensis*)	Material: peel Method: ultrasound-assisted extraction (45 kHz/1 h/45 °C) Solvent: H_2_O, EtOH, and hydroalcoholic (80% EtOH; 1:25 proportion) Separation: centrifugation (10,000× *g*/10 min)	Flavonoids: quantified (≠) Phenolic acids: (≠)	The extract exhibited TPC values (80%EtOH > H_2_O > EtOH) The extract exhibited FRAP and ABTS^•+^ values (H_2_O > 80%EtOH > EtOH)
Sirini et al. [54]	Kiwifruit peel (*Actinidia deliciosa*)	Method: ultrasound-assisted extraction (150–180 W/6 min) Solvent: hydroalcoholic (60% EtOH; 1:35 proportion) Separation: centrifugation (3398× *g*/5 min/22 °C) Concentration: under vacuum at 44 °C	Flavonoids: (≠) Phenolic acids: (≠)	The extract exhibited TPC, ABTS^•+^, and DPPH^•^ values

AOC, antioxidant capacity; EtOH, ethanol; MeOH, methanol; ø, conditions not reported; ≠, unidentified individual compound; TPC, total phenolic content; FRAP, ferric reducing antioxidant power method; ABTS^•+^, 2,2′-azino-bis(3-ethylbenzothiazoline-6-sulfonic acid); DPPH^•^, 2,2-diphenyl-1-picrylhydrazyl.

**Table 2:** AOC stability of natural ingredients subjected to sGD.

Reference	Natural Ingredient	Extraction of the Antioxidant Material	Bioactive Compounds Linked to AOC	Results of AOC After sGD
Zeng et al. [57]	Lychee (*Litchi* sp.)	Material: pericarp Method: maceration extraction (ø) Solvent: H_2_O (1:30 proportion)	Flavonoids: procyanidin B2, epicatechin, procyanidin A2, Quercetin-3-rutinose-7-rhamnoside, and isoquercitrin Phenolic acids: caffeic, ferulic	TPC and ABTS^•+^ values decreased, while FRAP values increased
Inada et al. [58]	Jaboticaba (*Plinia jaboticaba*)	Material: peel and seed Drying: freeze-drying (0.021 mbar/72 h/−51 °C)	Flavonoids: myricetin, myricetin-3-O-rhamnoside, quercetin, quercetin-3-O-rutinoside, cyanidin-3-O-glucoside, and delphinidin-3-O-glucoside Phenolic acids: gallic, m-coumaric, ellagic, and 3,4-dihydroxybenzoic	TPC values increased
Cao et al. [59]	Passion fruit (*Passiflora edulis*)	Material: peel Method: maceration extraction (ø) Solvent: hydroalcoholic (70% EtOH; 1:20 proportion) Separation: filtration (0.45 µm) Concentration: under vacuum (55 °C) Drying: freeze-drying (ø)	Flavonoids: apigenin 5-O-glucoside, rutin, and syringetin Phenolic acids: p-coumaraldehyde and 4-O-p-coumaroylquinic acid	TPC, FRAP, and DPPH^•^ values decreased
Bas-Bellver et al. [60]	Carrot (*Daucus carota* L.)	Material: whole waste Drying: freeze-drying (0.1 mbar/24 h/–45 °C)	Flavonoids: quantified (≠) Phenolic acids: (≠)	TPC and ABTS^•+^ values increased, while DPPH^•^ decreased
Cañas et al. [61]	Cocoa (*Theobroma* sp.)	Material: shell Method: maceration extraction (ø) Solvent: H_2_O (0.02 mg/mL; 90 min/100 °C) Separation: filtration (ø) Drying: freeze-drying at –20 °C	Flavonoids: apigenin-6,8-di-C-glucoside, catechin, epicatechin, quercetin 3-O-glucoside, and quercetin 3-O-arabinoside Phenolic acids: gallic and protocatechuic	TPC, FRAP, and ABTS^•+^ values increased (extract > powder)
Mercatante et al. [62]	Olive (*Olea* sp.)	Material: mill wastewaters Pre-treatment: enzymatic hydrolysis (12 h/20 °C) Drying: spray-dried (ø)	Flavonoids: (≠) Phenolic acids: (≠)	TPC and ABTS^•+^ values decreased
Wang et al. [63]	Pomegranate (*Punica granatum* L.)	Material: peel Method: ultrasound-assisted extraction (40 kHz/20 min/35 °C) Solvent: hydroalcoholic (70% MeOH; 1:50 proportion) Separation: centrifugation (8000× *g*/15 min)	Flavonoids: (≠) Phenolic acids: gallic and ellagic	TPC, FRAP, ABTS^•+^, and DPPH^•^ values were decreased
Wang et al. [64]	Jujube (*Ziziphus mauritiana*)	Material: peel and pulp Method: maceration extraction (ø) Solvent: H_2_O (1:5 proportion)	Flavonoids: naringenin tri-glycoside, quercetin-3-O-[(2-hexosyl)-6-rhamnosyl]-hexoside, quercetin 3-O-pentosylhexoside, and quercetin-3-O-(2-pentosyl-rhamnoside)-4′-O-rhamnoside Phenolic acids: (≠)	TPC, FRAP, and DPPH^•^ values increased
Elejalde et al. [65]	Grape (*Vitis* sp.)	Material: seed Method: ultrasound-assisted extraction (20 min) Solvent: hydroalcoholic (50% EtOH; 1:50 proportion) Separation: centrifugation (4000 rpm/5 min) and filtration (0.5 µ)	Flavonoids: catechin, epicatechin, epicatechin gallate, quercetin-3-glucoside, quercetin-3-rutinoside, malvidin-3-O-glucoside, delphinidin-3-O-glucoside, and cyanidin-3-O-glucoside Phenolic acids: (≠)	TPC and DPPH^•^ values increased
Soares et al. [66]	Yellow Puçá (*Mouriri grandiflora*)	Material: pulp, peel, and seed Method: ultrasound-assisted extraction (40 kHz/1 h/40 °C) Solvent: hydroalcoholic (80% EtOH; 1:4 proportion) Separation: filtration (ø)	Flavonoids: catechin Phenolic acids: gallic, chlorogenic, caffeic, p-coumaric, m-coumaric, o-coumaric, ferulic, rosmarinic, and trans-cinnamic	FRAP and DPPH^•^ values decreased

EtOH, ethanol; MeOH, methanol; ø, conditions not reported; ≠, unidentified individual compound; TPC, total phenolic content; FRAP, ferric reducing antioxidant power method; ABTS^•+^, 2,2′-azino-bis(3-ethylbenzothiazoline-6-sulfonic acid); DPPH^•^, 2,2-diphenyl-1-picrylhydrazyl.

**Table 3:** AOC of natural ingredient-treated uncooked meat products subjected to storage.

Reference	Natural Ingredient	Meat Product and Conditions	Extraction of the Antioxidant Material from the Uncooked Meat Product	Bioactive Compounds Linked to AOC	Results of AOC After Storage
Zajaç et al. [72]	Hyssop (*Hyssopus officinalis*) Borage (*Borago officinalis*)	Product: ground pork Addition: powder (0.5%) Storage: 15 days/4 °C	Material: whole plant Method: ultrasound-assisted extraction Solvent: EtOH (95%; 5 g of sample) Separation: centrifugation (3200× *g*/20 min) and filtration (Whatman No. 1)	Flavonoids: quercetin and rutin Phenolic acids: t-cinnamic, chlorogenic, p-coumaric, ferulic, caffeic, and hippuric	(↑) AOC (as measured by FRAP and DPPH^•^); [C] not demonstrated
Mancini et al. [18]	Turmeric (*Curcuma longa* L.)	Product: rabbit patties Addition: powder (3.5%) Storage: 7 days/4 °C	Method: maceration extraction Solvent: EtOH (9000 rpm/45 s; 5 g of sample) Separation: centrifugation (10,000 rpm/10 min) and filtration (Whatman No. 4)	Flavonoids: (≠) Phenolic acids: (≠)	(↑) AOC (FRAP, ABTS^•+^, and DPPH^•^); [C] not demonstrated
Hawashin et al. [73]	Olive (*Olea europea*)	Product: beef patties Addition: cake powder (2%, 4% and 6%) Storage: 6 days/4 °C	Method: maceration extraction Solvent: H_2_O (overnight/4 °C; 1:10 proportion) Separation: centrifugation (4500× *g*/30 min)	Flavonoids: (≠) Phenolic acids: (≠)	(↑) AOC (TPC and DPPH^•^); [C]
Ouerfelli et al. [74]	Neem (*Azadirachta indica* L.) Chilli (*Capsicum baccatum*)	Product: beef patties Addition: leaf powder (0.7%) Storage: 11 days/4 °C	Method: maceration extraction Solvent: H_2_O (5 g of sample) Separation: centrifugation (30 min/4 °C) and filtration (folded filter)	Flavonoids: (≠) Phenolic acids: (≠)	(↑) AOC (DPPH^•^) for both extracts; [C] not demonstrated
Ramírez-Rojo et al. [75]	Mesquite (*Prosopis velutina*)	Product: pork patties Addition: leaf H_2_O-EtOH extract (0.05% and 0.1%) Storage: 10 days/4 °C	Method: maceration extraction Solvent: H_2_O (1:10 proportion) Separation: centrifugation (4200× *g*/10 min/4 °C)	Flavonoids: (≠) Phenolic acids: (≠)	(↑) AOC (RPA and DPPH^•^); [C]
Vargas-Sánchez et al. [76]	Propolis	Product: beef and pork patties Addition: extract (2%) Storage: 9 days/4 °C	Method: maceration extraction Solvent: H_2_O (4500 rpm/1 min; 1:10 proportion) Separation: centrifugation (4200× *g*/10 min/4 °C)	Flavonoids: naringenin, quercetin, luteolin, kaempferol, apigenin, pinocembrin, pinobanksin 3-acetate, chrysin, galangin, acacetin, and pinostrobin Phenolic acids: gallic, cinnamic, and p-coumaric	(↑) AOC (TPC, RPA, and DPPH^•^) for both products; [C] not demonstrated
Al-Juhaimi et al. [77]	Baobab (*Adansonia digitata*)	Product: beef patties Addition: seed extract (2% and 3%) Storage: 21 days/4 °C	Method: maceration extraction Solvent: H_2_O (1:10 proportion)	Flavonoids: (≠) Phenolic acids: (≠)	(↑) AOC (TPC and DPPH^•^); [C] for TPC
Prommachart et al. [78]	Black rice (*Oryza sativa* L.)	Product: beef patties Addition: extract (0.4%, 0.8%, and 1.2%) Storage: 6 days/2 °C	Method: maceration extraction Solvent: MeOH (30 min/25 °C; 1:3 proportion) Separation: centrifugation (3000× *g*/15 min/4 °C)	Flavonoids: (≠) Phenolic acids: (≠)	(↑) AOC (DPPH^•^); [C]
Bellucci et al. [79]	Red pitaya (*Hylocereus monacanthus*)	Product: pork patties Addition: H_2_O extract (250, 500, and 1000 mg/kg) Storage: 18 days/2 °C	Method: maceration extraction Solvent: MeOH (1:20 proportion) Separation: centrifugation (10,000× *g*/10 min) and filtration	Flavonoids: (≠) Phenolic acids: (≠)	(↑) AOC (DPPH^•^); [C]
Vargas-Sánchez et al. [80]	Sun mushroom (*Agaricus brasiliensis*)	Product: pork patties Addition: H_2_O-EtOH extract (0.5% and 1%) Storage: 9 days/2 °C	Method: maceration extraction Solvent: hydroalcoholic (90%/4500 rpm/10 min; 1:10 proportion) Re-extraction: ultrasound-assisted extraction (42 kHz/1 h/25 °C) Separation: centrifugation (2300× *g*/10 min/4 °C) and filtration (0.22 µm)	Flavonoids: (≠) Phenolic acids: (≠)	(↑) AOC (TPC, RPA, ABTS^•+^, and DPPH^•^) for both products; [C]
Murillo-Hernández et al. [81]	Coffee (*Coffea* sp.)	Product: pork patties Addition: spent coffee grounds H_2_O-EtOH extract (0.5% and 1%) Storage: 9 days/4 °C	Method: maceration extraction Solvent: H_2_O (1:10 proportion) Separation: centrifugation (4500× *g*/10 min/4 °C)	Flavonoids: quantified (≠) Phenolic acids: (≠)	(↑) AOC (TPC, FRAP, RPA, ABTS^•+^, and DPPH); [C]
Vargas-Sánchez et al. [82]	Reishi (*Ganoderma lucidum*	Product: pork patties Addition: H_2_O-EtOH extract (0.5% and 1%) Storage: 9 days/2 °C	Method: maceration extraction Solvent: hydroalcoholic (50%/4500 rpm/1 min; 1:10 proportion) Re-extraction: ultrasound-assisted extraction (42 kHz/1 h/24 °C) Separation: centrifugation (2300× *g*/30 min/5 °C) and filtration (0.20 µm)	Flavonoids: chrysin Phenolic acids: gallic and protocatechuic	(↑) AOC (TPC, RPA, ABTS^•+^, and DPPH^•^) for both products; [C]
Ngongoni et al. [83]	Black wattle (*Acacia mearnsii*)	Product: pork patties Addition: bark and leaves (0.045%) Storage: 9 days/4 °C	Method: maceration extraction Solvent: PBS (10,000 rpm/30 s; 1:5 proportion) Separation: centrifugation (2575× *g*/10 min/4 °C) and filtration (Whatman No. 1)	Flavonoids: kaempferol 3- sophoroside, myricetin-3- rutinoside, myricetin-3- galactoside, myricetin 3- arabinoside, myricitrin, quercitrin, myricetin, epicatechin, catechin, and procyanidin A1-B1-B5, Phenolic acids: gallic, gentinsic, glucosyringic, and p-coumaroyltrifolin B	(↑) AOC (FRAP, ABTS^•+^, and DPPH^•^); [C] not demonstrated
Jin et al. [84]	Sappanwood (*Caesalpinia sappan*)	Product: pork sausage Addition: heartwood extract (0.05%, 0.1%, and 0.2%) Storage: 120 days/10 °C	Method: maceration extraction Solvent: EtOH (1:1 proportion)	Flavonoids: (≠) Phenolic acids: (≠)	(↑) AOC (DPPH^•^); [C]
Van Ba et al. [85]	Shiitake (*Lentinula edodes*)	Product: pork sausage Addition: H_2_O and EtOH extract (100 mL) Storage: 40 days/15 °C	Method: maceration extraction Solvent: H_2_O (1:1 proportion)	Flavonoids: (≠) Phenolic acids: (≠)	(↑) AOC (TPC and DPPH^•^)
Tran et al. [19]	Guava (*Psidium guajava* L.)	Product: pork sausages Addition: leaf extract (3000, 4000, 5000, and 6000 ppm) Storage: 14 days/4 °C	Method: maceration extraction Solvent: MeOH (1:4 proportion) Separation: centrifugation (3000× *g*/10 min/20 °C)	Flavonoids: (≠) Phenolic acids: (≠)	(↑) AOC (FRAP and DPPH); [C] for DPPH^•^

EtOH, ethanol; (↑), enhance; AOC, antioxidant capacity; [C], the effect depended on the concentration used; TPC, total phenolic content; FRAP, ferric reducing antioxidant power method; ABTS^•+^, 2,2′-azino-bis(3-ethylbenzothiazoline-6-sulfonic acid); DPPH^•^, 2,2-diphenyl-1-picrylhydrazyl.

**Table 4:** AOC of natural ingredient-treated cooked meat products subjected to storage.

Reference	Natural Ingredient	Meat Product and Conditions	Extraction of the Antioxidant Material from the Cooked Meat Product	Bioactive Compounds Linked to AOC	Results of AOC After Storage
Alnoumani et al. [86]	White button (*Agaricus bisporus*)	Product: ground beef Addition: powder (1%, 2%, and 4%) Storage: 16 days/4 °C	Method: maceration extraction Solvent: PBS (5 g of sample) Separation: centrifugation (127.8× *g*/15 min/4 °C) and filtration (cheesecloth)	Flavonoids: (≠) Phenolic acids: gallic, p-coumaric, and caffeic	(↑) AOC (TPC); [C]
Vargas-Sánchez et al. [80]	Sun mushroom (*Agaricus brasiliensis*)	Product: pork patties Addition: H_2_O-EtOH extract (0.5% and 1%) Storage: 9 days/2 °C	Method: maceration extraction Solvent: hydroalcoholic (90%/4500 rpm/10 min; 1:10 proportion) Re-extraction: ultrasound-assisted extraction (42 kHz/1 h/25 °C) Separation: centrifugation (2300× *g*/10 min/4 °C) and filtration (0.22 µm)	Flavonoids: (≠) Phenolic acids: (≠)	(↑) AOC (TPC, RPA, ABTS^•+^, and DPPH^•^) for both products; [C]
Vargas-Sánchez et al. [82]	Reishi (*Ganoderma lucidum*)	Product: pork patties Addition: H_2_O-EtOH extract (0.5% and 1%) Storage: 9 days/2 °C	Method: maceration extraction Solvent: hydroalcoholic (50%/4500 rpm/1 min; 1:10 proportion) Re-extraction: ultrasound-assisted extraction (42 kHz/1 h/24 °C) Separation: centrifugation (2300× *g*/30 min/5 °C) and filtration (0.20 µm)	Flavonoids: chrysin Phenolic acids: gallic and protocatechuic	(↑) AOC (TPC, RPA, ABTS^•+^, and DPPH^•^) for both products; [C]
Yim et al. [87]	Sappanwood (*Caesalpinia sappan*)	Product: pork sausage Addition: heartwood extract (0.007%, 0.0035% and 0.1%) Storage: 28 days/4 °C	Method: maceration extraction Solvent: H_2_O (1:2 proportion)	Flavonoids: (≠) Phenolic acids: (≠)	(↑) AOC (DPPH^•^); [C]
Bedrníček et al. [44]	Onion (*Allium* sp.)	Product: fish sausages Addition: powder (1%, 2%, and 3%) Storage: 28 days/5 °C	Method: maceration extraction Solvent: hydroalcoholic (90%; 1:10 proportion) Separation: centrifugation (7000 rpm/15 min/5 °C)	Flavonoids: quercetin, Quercetin-4-O-glucoside, and Quercetin-3,4-O-diglucoside Phenolic acids: (≠)	(↑) AOC (TPC, FRAP, and DPPH^•^); [C]
Manzoor et al. [88]	Mango (*Mangifera indica* L.)	Product: chicken sausages Addition: H_2_O peel extract (2%, 4%, and 6%) Storage: 10 days/4 °C	Method: maceration extraction Solvent: MeOH (1:33 proportion) Separation: centrifugation (10,000× *g*/10 min/4 °C)	Flavonoids: (≠) Phenolic acids: (≠)	(↑) AOC (DPPH^•^); [C]
Yaghoubi et al. [89]	Oleaster (*Elaeagnus angustifolia* L.)	Product: frankfurter-type sausages Addition: essential oil leaf (1000 and 2000 ppm) Storage: 45 days/4 °C	Method: maceration extraction Solvent: H_2_O (1:2 proportion)	Flavonoids: (≠) Phenolic acids: (≠)	(↑) AOC (TPC); [C]
López-Hernández et al. [30]	Cactus berry (*Myrtillocactus geometrizans*) Red prickly pear (*Opuntia ficus-indica* L.)	Product: chicken sausages Addition: powder in mixtures (75% + 25%, 25–75%, and 50–50%, respectively) Storage: 28 days/1.5 °C	Method: maceration extraction Solvent: EtOH (1:4 proportion) Separation: centrifugation (5000 rpm/15 min)	Flavonoids: quantified (≠) Phenolic acids: (≠)	(↑) AOC (FRAP, ABTS^•+^, and DPPH^•^)

EtOH, ethanol; MeOH, methanol; (↑), enhance; AOC, antioxidant capacity; [C], the effect depended on the concentration used; TPC, total phenolic content; FRAP, ferric reducing antioxidant power method; ABTS^•+^, 2,2′-azino-bis(3-ethylbenzothiazoline-6-sulfonic acid); DPPH^•^, 2,2-diphenyl-1-picrylhydrazyl.

**Table 5:** AOC of natural ingredient-treated cooked meat products subjected to gastrointestinal digestion.

Reference	Natural Ingredients	Meat Product and Conditions	Bioactive Compounds Linked to AOC	Results of AOC After sGD
Torres-Martínez et al. [97]	Oyster mushroom (*Pleurotus ostreatus*)	Product: pork patties Formulation: pork meat was replaced with back fat (10%), salt (1.5%), and the powder (0%, 2% and 5%; T1–T3).	Flavonoids: quantified (≠) Phenolic acids: (≠)	T2 and T3 showed the highest TPC, ABTS^•+^, and DPPH^•^ values, while T2 showed the highest FRAP values.
Ansorena and Astiasaran [98]	Rosemary (*Rosmarinus officinalis* L.) Parsley (*Petroselinum crispum*)	Product: ground beef patties Formulation: beef meat replaced with back fat (19.8%), salt (0.8%), and the extract (0% or 0.71; T1–T3)	Flavonoids: (≠) Phenolic acids: (≠)	T2 and T3 showed the highest DPPH^•^ values.
Lavado & Cava [99]	Pomegranate (*Punica granatum* L.)	Product: uncured dry sausages Formulation: pork meat was replaced with the aqueous extracts peel of each (0, 0.5, and 12.5 mg; T1–T3)	Flavonoids: quantified (≠) Phenolic acids: (≠)	T3 showed the highest FRAP, ABTS^•+^, and DPPH^•^ values.
Vargas-Sánchez et al. [82]	Reishi (*Ganoderma lucidum*)	Product: pork patties Formulation: pork meat was replaced with back fat (10%), salt (1.5%), and a hydroalcoholic extract (50%) (0%, 0.5% or 1%; T1–T3).	Flavonoids: chrysin Phenolic acids: gallic and protocatechuic	T2 and T3 showed the highest TPC values at the different concentrations used. T2 showed the highest DPPH^•^ values.
Khamzaeva et al. [100]	Soy (*Glycine max* L.) Wheat (*Triticum aestivum* L.)	Product: beef patties Formulation: pork meat was replaced with plant proteins (0% or 23%; T1–T3).	Flavonoids: (≠) Phenolic acids: (≠)	T3 showed the highest FRAP values.
Torres-Martínez et al. [101]	Oyster mushroom (*Pleurotus ostreatus*)	Product: minced pork Formulation: pork meat was replaced with back fat (20%), salt (1.5%), and water extract (0 or 500 µg/g; T1 and T2)	Flavonoids: quantified (≠) Phenolic acids: quantified (≠)	T2 showed the highest TPC, FRAP, ABTS^•+^, and DPPH^•^ values.

sGD, simulated gastrointestinal digestion. TPC, total phenolic content; FRAP, ferric reducing antioxidant power method; ABTS^•+^, 2,2′-azino-bis(3-ethylbenzothiazoline-6-sulfonic acid); DPPH^•^, 2,2-diphenyl-1-picrylhydrazyl.

## Data Availability

No new data were created or analyzed in this study. Data sharing does not apply to this article.

## Abbreviations

The following abbreviations are used in this manuscript:

AOC	Antioxidant capacity
AOH	Phenolic antioxidant
AO^•^	Phenoxyl radical
AOH^•^	Phenol radical cation
ABTS	2,2′-azino-bis(3-ethylbenzothiazoline-6-sulfonic acid
DPPH	2,2-diphenyl-1-picrylhydrazyl
ET	Single electron transfer
EtOH	Ethanol
FRAP	Ferric reducing antioxidant power
HAT	Hydrogen atom transfer
LOOH	Lipid hydroperoxide
LOO^•^	Lipid peroxyl radical
MeOH	Methanol
POOH	Protein hydroperoxide
POO^•^	Protein peroxyl radical
P−Fe^3+^	Protein-bound ferric ion
P−Fe^2+^	Protein-bound ferreous ion
sGD	Simulated gastrointestinal digestion
SETPT	Sequential electron-proton transfer
SPLET	Sequential proton-electron transfer
TPC	Total phenolic content
TPTZ	2,4,6-tris(2-pyridyl)-1,3,5-triazine acid
